# Autistic Traits in Treatment-Seeking Transgender Adults

**DOI:** 10.1007/s10803-018-3557-2

**Published:** 2018-04-13

**Authors:** Anna Nobili, Cris Glazebrook, Walter Pierre Bouman, Derek Glidden, Simon Baron-Cohen, Carrie Allison, Paula Smith, Jon Arcelus

**Affiliations:** 1 Nottingham National Centre for Transgender Health, Nottingham, UK; 2 Institute of Mental Health, University of Nottingham, Room B12, B Floor, Innovation Park, Triumph Road, Nottingham, NG7 2TU UK; 3 Nottingham City Asperger Service, Highbury Hospital, Nottingham, UK; 4 Autism Research Centre, Department of Psychiatry, University of Cambridge, Cambridge, UK

**Keywords:** Autism spectrum conditions, Autistic traits, Transgender, Autism spectrum quotient (AQ), Social issues, Social anxiety

## Abstract

The present study aimed to compare prevalence of autistic traits measured by the self-reported autism spectrum quotient-short (AQ-short) in a transgender clinical population (n = 656) matched by age and sex assigned at birth to a cisgender community sample. Results showed that transgender and cisgender people reported similar levels of possible autistic caseness. Transgender people assigned female were more likely to have clinically significant autistic traits compared to any other group. No difference was found between those assigned male. High AQ scores may not be indicative of the presence of an autism spectrum condition as the difference between groups mainly related to social behaviours; such scores may be a reflection of transgender people’s high social anxiety levels due to negative past experiences.

Transgender is an umbrella term used to describe individuals whose gender identity does not match their sex assigned at birth based on sexual characteristics. This includes people who do not identify within the binary gender of male and female (non-binary people) (Beek et al. 2015; Richards et al. 2017). Transgender women are those assigned male at birth but who identify as female whilst transgender men are individuals assigned female at birth, who identify as male (Bouman et al. 2017a). Some transgender people access transgender health services as they have a wish to transition medically to their experienced gender. These services include cross-sex hormone treatment (CHT) and referral for gender affirming surgeries, among other supports (Coleman et al. 2012).

Transgender people attending transgender health services experience high levels of mental health problems, including anxiety, depression and self-harm (Arcelus et al. 2016; Bouman et al. 2017b; Claes et al. 2015; Davey et al. 2016; Dhejne et al. 2016; Heylens et al. 2014; Millet et al. 2017). In addition, recent reports have hypothesised high levels of autistic traits amongst transgender people attending gender services (Glidden et al. 2016). However, the populations selected and the lack of appropriate comparison groups of cisgender participants (individuals whose experienced gender matches their sex assigned at birth) limit the results of many of these studies (Glidden et al. 2016; Strang et al. 2014; Shumer et al. 2015; Jacobs et al. 2014).

Literature on the prevalence of autistic traits showed that a large number of individuals displaying non-autistic psychopathology scored in the clinical range of ASC screening instruments without dealing with true ASC (Pine et al. 2008; Tonge et al. 2016; White et al. 2012). Despite these findings, there are strong clinical impressions suggesting a high prevalence of autistic traits in clinical transgender populations are supported by a number of published case studies looking at both children and adults and by a few quantitative studies (Kraemer et al. 2005; Lànden and Rasmussen 1997; Lemaire et al. 2014; Mukkades 2002; Parkinson 2014; Robinow 2009; Tateno et al. 2008; Vanderlaan et al. 2015). A recent systematic review exploring the frequency of autism spectrum disorder (ASD) or autism spectrum conditions (ASC) in transgender people suggested the presence of a relationship between being transgender and presenting with autistic traits in children and young people (Glidden et al. 2016). However, evidence to support the assumption of an excess of autistic traits in adult populations is still lacking (Jones et al. 2012; Pasterski et al. 2014).

Three adult studies assessed rates of autistic traits in the transgender populations. All three studies used versions of the autistic spectrum quotient (AQ) (Baron-Cohen et al. 2001). The AQ is a self-report instrument used to quantify autistic traits in adults of average or above average intelligence (Baron-Cohen et al. 2001). As the AQ-short is not a diagnostic tool, the definition of caseness might be misinterpreted as a diagnosis of this condition; thus caution is needed when interpreting the results (Hoekstra et al. 2008, 2011; Wheelwright et al. 2010).

AQ items are summed to give a total score, with suggested cut-offs for potentially clinically significant ASC. The scale measures two main higher order factors that related to represent areas describing either difficulties or core traits that are typically present in people with ASC; hence difficulties in social behaviours and a fascination with number and patterns. The first factor is further divided into subdomains relating to social skills and communication, attention switching and to detail, routine behaviours, and imagination.

Although there is no evidence that adult transgender populations as a whole have higher AQ scores compare to cisgender populations, the findings from all three studies suggest that transgender populations assigned female sex at birth may have elevated levels of autistic traits compared to cisgender females (Jones et al. 2012; Kristensen and Broome 2016; Pasterski et al. 2014).

Jones et al. (2012) compared autistic traits in a clinical, adult transgender sample with 61 transgender men (mean age 34 years) and 198 transgender women (mean age 45 years) to a cisgender (where the person’s gender matches the sex they were assigned at birth) control group (mean age 37 years) drawn from the general population (76 cisgender males, 98 cisgender females). Higher AQ scores in the transgender group were accounted for by a highly significant interaction between sex assigned at birth and transgender status. Transgender men (also referred to as female to male transgender people) had higher AQ scores compared to typical cisgender females with a large effect size (d = 1.0), but there was no difference between transgender women (also referred to as male to female transgender people) and typical cisgender males. Nearly 30% of transgender men had scores above 28 on the AQ putting them more than two standard deviations above a typical population mean and suggesting they fell within the autistic phenotype compared to only 2% of cisgender females. For transgender women the comparable rate was 5% and for cisgender males it was 6.6%.

Pasterski et al. (2014) compared 91 transgender individuals (63 transgender women and 28 transgender men; mean age 36.5 years) attending a transgender health service to a community control group. The study found no difference in total AQ scores between cisgender and transgender groups regardless of sex assigned at birth, but transgender males scored significantly higher in the social skills (d = 0.44) and attention switching (d = 0.68) sub-domains compared to cisgender females. These differences were not observed in transgender women using a validated cut-off of 32+ (Woodbury-Smith et al. 2005). Seven point one percent of transgender men had AQ scores that might indicate the presence of a clinical diagnosis of ASC compared to only 1% in cisgender women, although this difference was not significant. For transgender females and cisgender males the rate was 4.7 and 3.9% respectively.

Finally, an online survey through social media of 446 adults, who self-identified as transgender (Kristensen and Broome 2016) found that respondents assigned female sex at birth had significantly higher AQ scores as assessed by the brief AQ-10 (Allison et al. 2012) compared to transgender respondents assigned male sex at birth. This study found high rates of ASC, but did not include a cisgender comparison group. Nearly 40% of the sample scored above the AQ-10 cut-off, indicating risk for possible clinical ASC and 14% self-reported a clinical diagnosis of ASC (17% for assigned females at birth and 10% for assigned males at birth). These values are considerably higher than the prevalence of ASC in the general population, which is estimated at 1.5% for men and 0.2% for women (Brugha et al. 2011).

Therefore, the existing evidence suggests that the high rates of autistic traits found in studies including adult transgender people appears to apply to those assigned a female sex at birth (Jones et al. 2012). This may be explained by the extreme male brain theory (EMB) (Baron-Cohen 2002), which suggests that a higher number of autistic traits is associated with exposure to higher levels of foetal testosterone (Auyeung et al. 2009; Baron-Cohen et al. 2015). Affected individuals may therefore have more traits associated with a ‘male brain’ such as an increased drive to systemize and lower levels of empathy (Baron-Cohen 2003). Only one study has used the AQ to investigate which specific autistic traits are particularly prominent in transgender populations (Pasterski et al. 2014). This study did not exclude participants who had received cross-sex hormone therapy before their first assessment at the transgender health service. As cross-sex hormone therapy reduces anxiety this may have a direct effect on social skills (e.g. increase in social confidence and social functioning, experiencing fewer problems with socialisation and less social distress), one of elevated autistic traits (Bouman et al. 2016a, 2017b; Gómez-Gil et al. 2012). Other limitations in the small existing literature are lack of an adequate cisgender comparison group and small, poorly matched samples.

In view of the above limitations and in order to investigate whether there is any validity to clinicians’ impressions of higher autistic traits among the adult transgender population attending transgender health services, this study aims to compare the frequency of autistic traits in a large representative sample of patients attending a transgender health service to a large cisgender community population matched by age and sex assigned at birth. Additionally, the study aims to compare the profile of autistic traits between the two samples stratified by sex assigned at birth. Based on previous research (Jones et al. 2012; Pasterski et al. 2014), it is hypothesised that transgender participants assigned female at birth will have higher total scores on a measure of autistic traits compared to cisgender females.

## Methods

### Participants

Transgender group: All patients (n = 1020) invited to attend an initial assessment at a national adult transgender healthcare service in the UK between November 2012 and July 2016 were invited to participate in the study. Of these, 1002 (98.2%) consented. People on cross-sex hormone previous to assessment were excluded (n = 293) and data were missing from 48 people. Therefore a total of 661 (69.6%) were included in the study and eligible for matching.

Cisgender group: The control group consisting of cisgender individuals was drawn from a community sample recruited through the Cambridge Psychology (http://www.cambridgepsychology.com) website. This is a general psychology research website for adults, which aims at recruiting individuals in the general population who wish to take part in research studies. People become aware of this website by word of mouth and online searches. Participants who self-identified as transgender were excluded from the control group. Thus, a total sample consisting of 4070 cisgender people was considered for matching.

### Settings

Transgender participants were recruited from the Nottingham Centre for Transgender Health in the UK. This is a nationally commissioned service for people living in England and Wales. The centre is one of the largest transgender health clinics in Europe, receiving more than 1000 referrals annually of people over the age of 17 years. The transgender health clinic’s treatment programmes incorporate assessment for treatment suitability, psychological support (if needed), cross-sex hormone therapy and provides patients with referrals for gender affirming surgeries.

### Design

A case–control design was used for this study. Transgender participants were matched with cisgender participants 1:1 for age and sex assigned at birth by a researcher (AN) blind to AQ score. Of 661 eligible participants in the transgender group it was possible to find 656 sex assigned and age matched controls from the cisgender sample. Participants were matched by sex assigned at birth in order to not exclude non-binary identities, when matching to the cisgender group.

### Main Outcome Measures

#### Autism Spectrum Quotient Short Version—AQ-Short (Hoekstra et al. 2011)

The AQ-short is a self-administered 28-items scale designed to measure autistic traits in individuals with normal intelligence and thus to give an indication of where a person lies on the continuum of the spectrum, ranging from healthy to autistic (Woodbury-Smith et al. 2005). The AQ-short is a shortened form of the well-validated AQ-50 (Baron-Cohen et al. 2001) and was developed by selecting the 28 most relevant items with the aid of factor analysis. It comprises two higher order factors related to autistic traits, including numbers and patterns, which assesses the extent to which people are fascinated by numbers, dates, patterns and categories, and social behaviours. The social behaviours factor represents skills related to social functioning and it comprises four subscales: (1) social skills, which assesses abilities and struggles in social interactions, (2) routine, which assesses the extent to which people use routine to manage new situations, (3) switching, which assesses how easily people can move from one demand to another as well as attention switching difficulties, and (4) imagination, which assesses the extent to which people can understand others’ perspectives, intentions as well as to create mental pictures (also known as empathic imagination). The latter subscale is also related to theory of mind (ToM) (Baron-Cohen 1991). Hence, the AQ-short captures difficulties or core traits typically found in people with autism, such as adapting to change, developing less rigid mental and behavioural flexibility as well as social skills (Hoekstra et al. 2008). Like the AQ-50, the AQ-short utilises a 4-point Likert scale ranging from “definitely agree” to “definitely disagree”. Item scores were reversed as appropriate so that higher values equated to more autistic traits. Total scores range between 28 and 112. Although not intended to be diagnostic tool, a cut-off of ≥ 70 was found to have a sensitivity of 0.94 and specificity of 0.91 to discriminate between an autism sample and a community sample. Cronbach’s alphas ranged between 0.77 and 0.86 (Hoekstra et al. 2011).

### Procedure

**Fig. 1:**
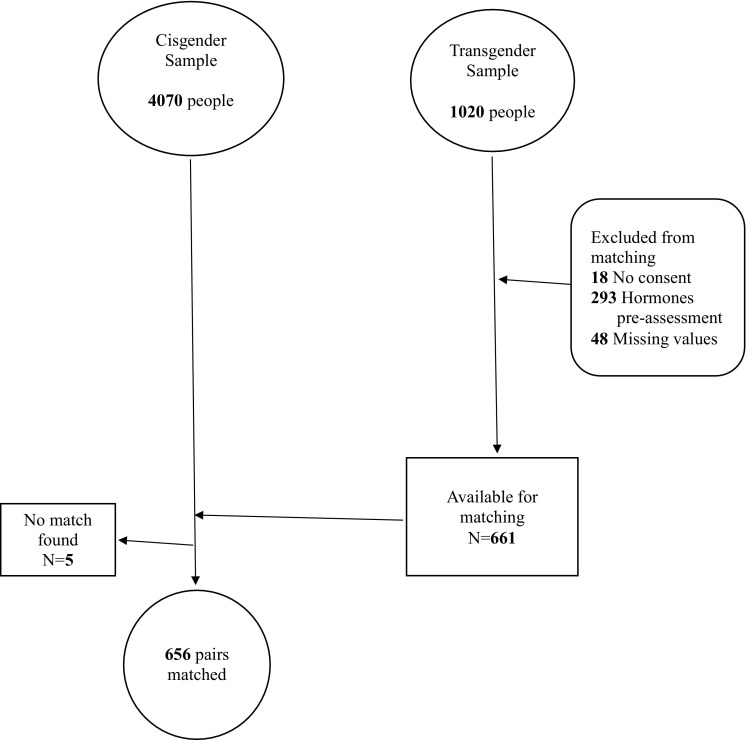
Flow chart of exclusion and matching procedures

Transgender participants were invited to complete a series of questionnaires as part of a longitudinal study prior to the first assessment appointment. The questionnaire package included socio-demographic information and the AQ-short. Information regarding cross-sex hormone treatment was also collected. Cisgender participants completed an online version of the AQ-50 and provided socio-demographic information. AQ-short items were extracted from the AQ-50 for the cisgender group (Hoekstra et al. 2011) and total AQ-short scores, factor scores and subscale scores were calculated for each participant. Participants in the transgender group meeting inclusion and exclusion criteria were matched blind 1:1 by age and sex assigned at birth with participants in the control group (see Fig. 1).

Ethical approval was received for the study from the NHS Ethics Committee (14/EM/0092) and by the Research and Development Department of the Nottinghamshire Healthcare NHS Foundation Trust in line with the Health Research Authority guidance (HRA 2013).

### Data Analysis

Data analyses were performed using the Statistical Software Package SPSS 23 (IBM 2015). First, frequencies and descriptive statistics were applied to the samples. As the variables were normally distributed, parametric analyses were performed. Chi square analysis was used to compare possible ASC caseness (AQ-short ≥ 70) between the groups and odds ratios calculated to express the likelihood of possible ASC caseness occurring in one group compared to another (Field 2009). Paired t-tests were used to compare AQ-short scores between groups. Independent t-tests were used to ascertain differences in groups between assigned females and assigned males at birth. An analysis of variance (ANOVA) with age as a covariate was carried out to explore potential interactions between group and sex assigned at birth. Finally, Cronbach’s alphas were executed to determine if the AQ-short is a reliable measure for use with transgender populations attending clinical services. The level of significance used for the statistical analyses was p < 0.05.

## Results

### Socio-demographic Characteristics of the Matched Samples

A total of 1312 people participated in the study (656 transgender and 656 cisgender). The mean age for each group was 28.28 years (SD = 12.25, range 16–74 years). A total of 260 people (39.6%) in each group were assigned female at birth whilst 396 (60.4%) were assigned male. There was a significant difference in employment status between the groups (χ^2^ = 144.85, df = 3, p < 0.001) with more unemployed participants in the transgender group and more students in the cisgender group (Table 1).

**Table 1:** Descriptive statistics for the transgender and cisgender groups

Variable	Transgender [M ± SD] or [N (%)] N = 656	Cisgender [M ± SD] or [N (%)] N = 656
Mean age (SD)	28.25 ± 12.25	28.25 ± 12.25
Assigned females at birth (%)	260 (39.6)	260 (39.6)
Assigned males at birth (%)	396 (60.4)	396 (60.4)
Employment status*		
Employed	114^a^ (37.6)	262^b^ (43)
Unemployed	80 (26.4)	17 (2.8)
Student	64 (21.1)	275 (45.1)
Other	45 (14.9)	56 (9.2)

*p < 0.001

^a^353 not asked for employment status, 33 missing values

^b^39 missing values

### Differences in Clinical Caseness Between Groups

Within the cisgender group 218 people (33.2%) were found to have scores at or above 70, indicating possible ASC caseness, compared to 238 (36.3%) in the transgender group (Table 2). The difference between the groups was not statistically significant (χ^2^ = 1.344, df = 1, p = 0.271).

**Table 2:** ASD cases reaching clinical significance by group and sex assigned at birth [N (%)] (n = 1312)

Group	Transgender [N (%)] N = 656	Cisgender [N (%)] N = 656
Total sample		
ASD caseness	238 (36.3)	218 (33.2)
Non ASD caseness	418 (63.7)	438 (66.8)
Assigned females at birth (n = 520)		
ASD caseness	118 (45.4)	78 (30)
Non ASD caseness	142 (54.4)	182 (70)
Assigned males at birth (n = 792)		
ASD caseness	120 (30.3)	140 (35.4)
Non ASD caseness	276 (69.7)	256 (64.6)

Confidence interval = 95%

*p < 0.01

When comparing participants assigned female sex at birth, a statistically significant difference was found between groups with 45% of the transgender group scoring ≥ 70 in total AQ scores, compared to 30% in the cisgender group (30%) (χ^2^ = 13.102, df = 1, p < 0.001). This represents nearly twice the risk of scoring above the screening cut-off (OR = 1.939, 95% CI 1.35–2.78, p < 0.001) for the transgender group. There was no difference between groups for assigned males at birth (χ^2^ = 2.29, df = 1, p = 0.15).

### Differences in AQ-Short Total and Subscale Scores Between Groups

Taking the group as a whole, the transgender group had statistically significantly lower AQ-short total scores compared to cisgender people [*t*(1188) = 1.96, p = 0.05, two tailed], but the effect size was small (d = 0.11). The transgender group had slightly, but statistically significantly higher scores for the higher order factor of Social Behaviour (p = 0.004) and lower scores for the higher order factor of numbers and patterns (p < 0.000). Analyses of the Social Behaviour subscale scores found that the transgender group had higher scores, indicating more difficulties with the social skills, routine and switching. However, they reported lower scores for Imagination subscale and the higher order factor of numbers and patterns suggesting fewer problems with understanding the perspective of others and less preoccupation and/or fascination with logical sequences related to numbers and patterns (Table 3).

**Table 3:** T-test results comparing transgender and cisgender people on AQ-short subscales and total scores (M ± SD) (n = 1312)

AQ-short subscales	Transgender N = 656	Cisgender N = 656	*t*	df	Effect size Cohen’s d
Social behaviours	54.95 ± 10.72	53.52 ± 7.11	2.85*	1137.562	0.16
Social skills	17.75 ± 4.9	16.65 ± 2.77	4.951**	1029.204	0.28
Routine	10.77 ± 2.71	9.12 ± 2	12.565**	1204.599	0.69
Attention switching	10.09 ± 2.54	9.09 ± 2.33	7.446**	1300.088	0.41
Imagination	16.34 ± 4.23	18.65 ± 3.12	11.28**	1205.385	− 0.62
Numbers and patterns	10.82 ± 3.4	13.36 ± 3.52	13.31**	1310	− 0.73
Total AQ-short	65.77 ± 11.81	66.88 ± 8.48	1.96*	1188.384	− 0.11

*p ≤ 0.05, **p < 0.001

### AQ-Short Total and Subscale Scores by Assigned Gender at Birth

**Fig. 2:**
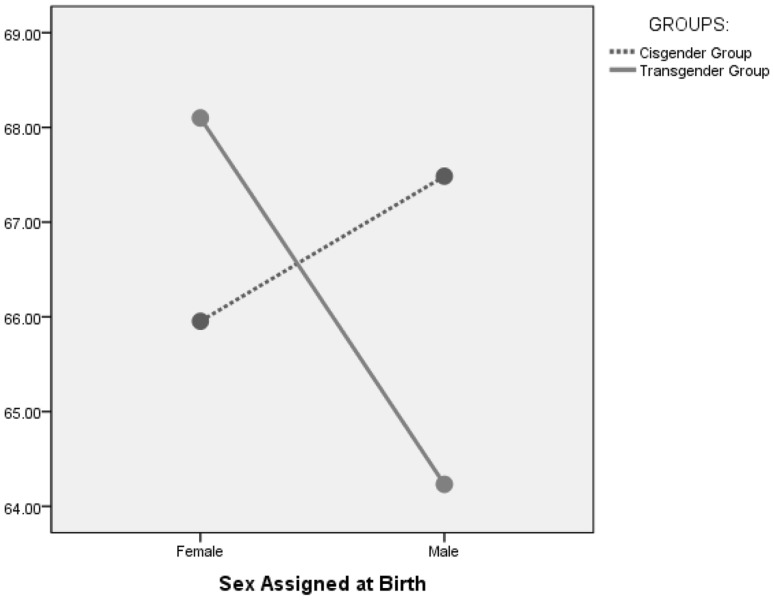
Estimated marginal means of AQ-short total scores for transgender and cisgender groups, by sex assigned at birth

To test the hypothesis that higher levels of autistic traits are found in transgender participants who were birth assigned females compared to cisgender females, a two way ANOVA was carried out with total AQ-short as the dependent variable and sex assigned at birth (male/female) and group (transgender/cisgender) as the independent factors. Age was included as covariate. There were no main effects of sex assigned at birth or group, but there was a statistically significant interaction between the variables [F(11307) = 22.006, p < 0.001] (Fig. 2). The covariate was not significant [F(11307) = 0.195, p > 0.05] indicating that age was unrelated to AQ-short total scores. Post hoc analyses found that birth assigned females in the transgender group had higher total scores (d = 0.2, p = 0.22) compared to the cisgender group, whilst for birth assigned males the cisgender group had higher scores (d = − 0.32, p < 0.001) (Table 4).

**Table 4:** AQ-short total and subscale scores by group and birth assigned gender (M ± SD) (n = 1312)

AQ-short subscales	Birth assigned females (n = 520)				Birth assigned males (n = 792)			
	Trans n = 260	Cis n = 260	t (df)	Effect size Cohen’s d	Trans n = 396	Cis n = 396	*t* (df)	Effect size Cohen’s d
Social behaviours	57.26 ± 11.26	52.76 ± 6.33	5.809 (259)**	0.49	53.43 ± 10.08	54.02 ± 7.54	− 0.937 (395)	− 0.05
Social skills	18.76 ± 5.1	16.44 ± 2.64	6.687 (259)**	0.57	17.08 ± 4.74	16.79 ± 2.85	1.063 (395)	0.07
Routine	11.19 ± 2.76	8.87 ± 1.98	10.994 (259)**	0.97	10.5 ± 2.64	9.29 ± 1.99	7.488 (395)**	0.52
Attention switching	10.53 ± 2.62	9.17 ± 2.19	6.359 (259)**	0.56	9.8 ± 2.45	9.03 ± 2.42	4.368 (395)**	− 0.31
Imagination	16.78 ± 4.48	18.27 ± 2.91	− 4.529 (259)**	− 0.39	16.05 ± 4.03	18.9 ± 3.23	− 10.741 (395)**	− 0.78
Numbers and patterns	10.78 ± 3.51	13.23 ± 3.65	− 7.804 (259)**	− 0.66	10.84 ± 3.33	13.44 ± 3.44	− 10.838 (395)**	− 0.77
Total AQ-short	68.05 ± 12.53	65.99 ± 7.84	2.305 (259)*	0.2	64.27 ± 11.08	67.46 ± 8.84	− 4.478 (395)**	− 0.32

*p < 0.05; **p < 0.001

Analysing the factors separately suggested that higher AQ-short scores for the transgender group within the birth assigned females were due to substantially higher scores for social behaviour. For assigned males there was no significant difference in social behaviour scores between the groups. For numbers and patterns the cisgender group had higher scores regardless of gender. In terms of subscales, there was a significant group difference in social skills for birth assigned females, but not birth assigned males. For other subscales the pattern of differences was similar for birth assigned males and birth assigned females, with higher scores in the transgender group for Routine and Switching and higher sores in the cisgender gender group for Imagination and numbers and patterns (Table 4).

Finally, Cronbach’s alphas for internal consistency were carried out to investigate if the AQ-short is a reliable measure for use with transgender populations. The AQ-short scale was found to have good internal consistency for the transgender group (28 items; α = 0.845) and acceptable internal consistency with the cisgender sample (28 items; α = 0.674).

## Discussion

Clinical reports and previous studies have suggested the existence of high levels of autistic traits among transgender people attending clinical services seeking gender affirming medical interventions (Glidden et al. 2016). Although previous studies have investigated levels of ASD in children attending gender identity services (de Vries et al. 2010) very few have focused on the adult population. Those studies recruiting adult transgender people, whether via clinical services or online surveys, have reported high levels of autistic traits and diagnoses (Kristensen and Broome 2016; Pasterski et al. 2014). However, those studies have failed to compare their results with a matched controlled population of cisgender people. The need for matching in this field is particularly important as autistic traits are higher on average amongst males (Baron-Cohen et al. 2001; Baron-Cohen 2002). Therefore, with the aim of investigating the levels of autistic traits in the transgender population attending services and whether those traits were different to a matched cisgender group, this study recruited a large population of treatment-seeking transgender adults and compared them to a large cisgender control group.

The study found that when comparing both groups there were no significant differences in the number of people who presented with scores suggesting a possible diagnosis of ASC. In fact, the levels of possible caseness of ASC was found to be high in both groups, with nearly a third of participants in both the transgender (36.3%) and the cisgender (33.2%) groups scoring above the clinical cut-off for possible ASC caseness. The findings of this study suggest higher prevalence rate of possible ASD cases among transgender individuals than some studies (Pasterski et al. 2014), but lower than others (Jones et al. 2012; Kristensen and Broome 2016). The sampling techniques and different cut-offs used by previous studies make direct comparisons difficult. However, the general clinical impression of an over-representation of people displaying autistic traits among transgender clinical populations when compared to cisgender people from the general population (Glidden et al. 2016) was not confirmed by the findings of the present study.

This study confirmed previous findings from adult studies that there is no evidence of increased rates of autism in transgender populations as a whole. It should be noted that the number of possible ASC caseness in both cisgender and transgender people is significantly higher than the prevalence of ASC which has been estimated at 1.1% in the general population (Brugha et al. 2011). Thus, the large number of cisgender people with possible ASC may have affected the results of the study. A possible explanation may be that the AQ demonstrates a stronger negative predictive value for ASC than a positive predictive value in the samples. The AQ being a screening tool rather than a diagnostic tool fits with this interpretation. Studies investigating levels of ASD in the general population have found similar rates of clinical ASD caseness (Kristensen and Broome 2016). Hoekstra et al. (2011) developed and validated the AQ-short and found that both UK and Dutch control samples reported average AQ-short total scores of approximately five points lower than our cisgender sample.

It was when exploring the results according to gender that the findings become more significant. Although no differences were found between people assigned male at birth (possible transgender female and/or non-binary people) and cisgender males, a significant difference was found when comparing people assigned female at birth. The study found that within people assigned female at birth the transgender group (possible transgender males and/or non-binary people) was about twice as likely to have clinically significant levels of autistic traits compared to cisgender females. This is in keeping with the findings of previous research using different versions of the same measure (AQ) (Jones et al. 2012; Kristensen and Broome 2016; Pasterski et al. 2014). With regard to the cisgender sample the findings of the present study appear to support previous research suggesting the presence of a male bias in the presentation of autistic traits (i.e., EMB theory) (Baron-Cohen et al. 2001; Baron-Cohen 2002; Hoekstra et al. 2008, 2011).

With regard to the transgender sample, the present results suggesting that transgender assigned females at birth suffer from higher overall autistic traits than transgender assigned males at birth are partially in line with findings of previous research (Kristensen and Broome 2016; Jones et al. 2012). A possible explanation for this might still be the abovementioned EMB theory (Baron-Cohen et al. 2001), which posits that assigned males at birth are more likely to and have the tendency to suffer from higher levels of autism. Consequently, transgender men might also face more social difficulties and worries related to having to change gender-roles and thus gender-specific behaviours (e.g. not feeling masculine enough, fear of being perceived as female), which might lead to experiencing higher levels of social anxiety. These results are in conflict with past literature suggesting higher levels of social anxiety in transgender women due to the reduced social acceptance of gender variant behaviours amongst assigned males than assigned females (e.g. society being more accepting of masculinity than femininity) (Yu et al. 2017). Future research should try to properly understand the different impact of autistic traits onto transgender assigned females as well as on assigned males at birth.

There is a feeling amongst clinicians that current screening and diagnostic tools may be biased towards identifying and diagnosing assigned males. As a consequence, assigned females have been under diagnosed by current clinical practice (Gould and Ashton-Smith 2011; Lai et al. 2011; Brugha et al. 2016).

The present study also examined AQ-short subscale scores to understand differences in autistic traits between groups. The study found that there were differences in the autistic traits between groups. Transgender people (particularly those assigned female at birth) were found to have specific difficulties in relation to social behaviours, including social skills, mental flexibility and problems switching attention, the disruption of which could lead to increments in anxiety levels (Batten 2005). These findings could be associated with social anxiety, which is known to be highly prevalent among the transgender population (Millet et al. 2017). The transgender group, however, reported fewer difficulties in relation to other autistic traits of poor imagination, and fascination with numbers and patterns. Therefore, there may be a confounding element where the transgender sample scored higher on the AQ without the presence of ASC. This may indicate that some transgender people do not really present with ASC but the high levels of social difficulties due to anxiety, depression and years of victimisation may affect the way they interact with others, as suggested by Turban and van Schalkwyk (2018). In fact interpersonal difficulties among this population have already been described (Davey et al. 2016). However, this would not explain higher scores on attention switching or attention to detail.

Enhanced scores on the subscales for routine and attention switching in the transgender population might be linked to the elevated social anxiety symptomatology experienced by this population. In fact, previous research has suggested that autistic individuals report similar scores on the attention switching subscale of the AQ-50 to people with a diagnosis of social anxiety disorder (Cath et al. 2008). They also stated that socially anxious people have been found to display higher scores than non-clinical controls (Cath et al. 2008). Thus, high scores on the routine subscale might reflect an increase in social anxiety levels due to the social difficulties related to changes in routine behaviours (e.g. switching gender roles, which imply learning and practicing different gender-specific behaviours), whilst high scores on the attention switching subscale might indicate difficulties in altering the focus of attention from negative and anxiety-producing experiences (e.g. being looked at in the street might lead to an increase in social anxiety levels due to the transgender individual’s fear of being recognised as trans) to more positive and adaptive coping behaviours (e.g. taking into consideration that being looked at in the street might be due to reasons other than the person being recognised as transgender). Transgender people are known to face these social challenges during the time of transition (Grossman and D’augelli 2006), which might result in them scoring higher than cisgender people on the subscales related to such difficulties.

As suggested by Lombardi (2001), transgender individuals’ extreme marginalisation and vulnerability might lead them to experience increased psychological, health and especially social isolation, when compared to other social groups. Consequently, some transgender people might score particularly high on some AQ-short items and subscales because of their dysphoria and related anxiety as well as negative past experiences (e.g. transphobia, bullying) (Skagerberg et al. 2015). Thus, certain scores on the AQ-short might simply indicate increased social difficulties, which would increase their AQ-short total scores reaching an ASD caseness level, while not being driven by the presence of ASC. This idea is supported by literature investigating autistic traits in socially anxious populations with the use of both AQ-50 (White et al. 2012) and AQ-short (Tonge et al. 2016). These studies suggested that the scores in the subscales related to social functioning and behaviours might be inflated by the difficulties experienced in social interactions and that scores on the AQ subscales related to social functioning need to be interpreted with caution when the subjects display marked social difficulties (Tonge et al. 2016; White et al. 2012). Against this, studies recruiting those with social anxiety may well be sampling individuals who have higher levels of autistic traits. It will be important to try to disentangle these in the future.

Notwithstanding the strengths of the present research (e.g., large sample size, matched controls, and homogeneity of sample in terms of treatment status), there are several limitations. First, as a case–control study was adopted participants should have been matched on additional variables other than age and sex assigned at birth (e.g., IQ, educational level) to properly eliminate the issue of confounding and gaining appropriate efficiency. Second, as previously discussed the AQ-short is a self-reported assessment tool for autistic traits instead of a diagnostic tool. Third, the data relative to the cisgender participants was gained through the “Cambridge Psychology” website and snowballing sampling and response bias might have biased the results as high levels of autistic traits were found in this population. Fourth, transgender and cisgender groups completed different versions of the AQ, which might have had an impact on how the participants approached and answered the items of the scale, although the items collected from the AQ-50 were the same as per the AQ-short.

Future studies may focus on analysing the role of gender affirming treatment (cross-sex hormones) in autistic traits as measured by the AQ. It has been noted clinically that transgender people with a previous diagnosis of ASC express fewer and less obvious autistic traits following medical transition. This has raised questions about the diagnostic safety of an ASC diagnosis made in transgender people. Studies which include diagnostic tools such as a clinical interview and/or formal diagnostic tools such as the Autism Diagnostic Interview Revised (ADI-R—Lord et al. 1994), Autism Diagnostic Observation Schedule (ADOS—Lord et al. 2002) and Diagnostic Interview for Social and Communication Disorders (DISCO—Wing et al. 2002) and which control for transitional status and anxiety are needed to fully explore the nature of elevated autistic traits in transgender populations.

Overall, this study found that autistic traits appear to be more prevalent in transgender people assigned female at birth, but not in those assigned male at birth. The autistic traits found in both groups appear to be particularly connected to social skills, which may be associated to the high levels of anxiety and low self-esteem that this group often experiences (Claes et al. 2015; Bouman et al. 2016b, 2017b; Millet et al. 2017). As transgender people displayed issues with the social features of ASC whilst reduced difficulties with autistic traits that may be less likely to be mimicked by the experience of gender dysphoria, undergoing gender affirming medical treatment might have a positive impact by improving interpersonal skills and social interactions. Longitudinal studies investigating the role of cross-sex hormone treatment on autistic traits are therefore needed as they may help reaching more robust evidence as to whether the observed autistic traits equate to clinical ASC or not.
